# Supplementary material for: Enhancing tertiary students’ programming skills with an explainable Educational Data Mining approach
Source: PLoS One. 2024 Sep 3;19(9):e0307536. doi: 10.1371/journal.pone.0307536 (PMC11371252; doi:10.1371/journal.pone.0307536)
Supplement: Attachment — Submitted filename: PLOS_ONE_EDM_Response_letter.pdf [file pone.0307536.s001.pdf]

Summary of Revisions of PONE-D-24-08321

Title: Enhancing Tertiary Students' Programming Skills: An Improved and Explainable Educational Data Mining Approach

Authors: Md Rashedul Islam, Adiba Mahjabin Nitu, Md Abu Marjan, Md Palash Uddin, Masud Ibn Afjal, and Md Abdulla Al Mamun

First, we would like to sincerely thank the editor and anonymous reviewers for their precious time and insightful comments on our manuscript. We have addressed all the comments made by the reviewers and revised the manuscript accordingly. In this document, we elaborate on how we have addressed the concerns of the reviewers. For the convenience of the editor and reviewers, the changes are highlighted in red color in the revised manuscript.

To Reviewer 1

Comment 1: The study was conducted on tertiary students' programming skills and their classification. Well-known ML methods, an ensemble model and XAI methods, which have been a hot topic in recent years, were applied on a dataset of 1720 data. The reviewer's evaluation of the paper is as follows; Successful points of the paper: - It is nice that both classical ML and current methods are used and presented comparatively. - SMOTE and NearMiss preprocessing performed on the imbalanced data set is correct - The outputs of the Global and Local methods are given successfully.

Response 1: Thanks to the reviewer for the insightful evaluation of our previous manuscript.

Comment 2: Weaknesses and Criticisms of the Paper. The originality of the article is unfortunately weak. It mostly consists of applying existing methods to a data set and sharing the results obtained by performing classical data mining steps. It could have been better presented what exactly the authors were aiming for with this study

Response 2: Thanks for the constructive feedback. We appreciate the opportunity to clarify the originality and objectives of our study. Our study introduces several innovative aspects, as follows: (i) Customized Algorithmic Enhancements: We proposed an ensemble Machine Learning (ML) approach after specific modifications to existing ML algorithms to improve the performance and applicability for predicting and enhancing tertiary students' programming skills in an educational context; (ii) Integration of Explainable AI (XAI): We incorporated XAI techniques to provide transparency and interpretability in the educational data mining (EDM) process that poses a significant advancement over traditional black-box models; (iii) Comprehensive Evaluation Framework: Our evaluation framework included multiple performance metrics and cross-validation techniques to ensure the reliability and generalizability of our results; and (iv) Actionable Insights for Educators: We offer practical insights and recommendations for educators that directly impact teaching strategies and student support mechanisms. We have now comprehensively incorporated the above discussion in the introduction section (**page#2**) of the revised manuscript to better highlight these novel

innovations and the specific objectives of our study. We believe these enhancements underscore the originality and significance of our work.

Comment 3:- It would have been better if the data sets preferred cross-validation instead of using three different train-test rates

Response 3: We sincerely appreciate your constructive feedback. In response to your suggestion, we have now employed 10-fold cross-validation for all ML algorithms used in our study. Please refer to the newly added **Section 4.5 (page#15) and Table 13 (page#15)** to get this updated result analysis in the revised manuscript.

Comment 4:- It would be better to use FFNN or some deep models instead of ANN. ANN is more suitable for simple classifications.

Response 4: Thank you for your insightful comment. We want to clarify that in our experiments, our implementation of ANN utilized a Feedforward Neural Network (FFNN) as the deep learning model. This approach allows us to leverage the strengths of deep learning for complex classification tasks. We have ensured that this clarification is explicitly mentioned in **Section 3.4 (page#8-9)** of the revised manuscript to avoid any confusion.

Comment 5:- It is unnecessary to present the results given in tables as graphs.

Response 5: Thank you for your insightful feedback. Based on your suggestion, we have removed the redundant graph (Fig. 2 of the previous manuscript) that duplicated the information already presented in the table.

Comment 6:- Especially XAI graphics resolutions should be improved.

Response 6: Thank you for your valuable feedback. We have updated the resolutions of the XAI graphics in the revised manuscript (**Section 5, page#17-19**) to convey the insights generated by our XAI module more effectively. Please note that we will also perform any further modifications to any figures' quality to make them compatible with the journal requirements, once accepted.

Comment 7:- Literature research seems to be insufficient. It needs to be improved. It would be useful for the authors to update this section in a more academic style with critical or innovative aspects. There are no interpretable meta-heuristic approaches in XAI methods (Section 3.5). Adding the following new approaches to the relevant section may offer a broader perspective to the readers. a. https://www.sciencedirect.com/science/article/abs/pii/S0925231220309954 b. https://www.sciencedirect.com/science/article/abs/pii/S0925231222008220

Response 7: We have now updated the literature review section (**Section 2, page#2-3**) in the revised manuscript by highlighting critical or innovative aspects of the existing works and adding a recent related work to the discussion and **Table 1 (page#3)** as well (Refn. [20]). Furthermore, according to the reviewer's suggestion, we have incorporated additional experimental result analysis using the Grey-Wolf-Optimizer as an interpretable metaheuristic XAI tool in the revised manuscript (**Section 3.6.1 (page#11), Section 5.1 (page#17), and Fig. 5 (page#17)**) and cited those above-mentioned two related articles.

To Reviewer 2

Comment 1: Strengths

Innovative Approach: The introduction of the Stacking-SRDA ensemble method and the use of XAI tools like shapash, eli5, and LIME are commendable for enhancing both the accuracy and transparency of the predictions.

Comprehensive Evaluation: The manuscript rigorously evaluates the proposed system using six different machine learning algorithms across various metrics like accuracy, precision, recall, and f1-score, which enhances the credibility of the results.

Practical Implications: The development of a system for identifying skill gaps in programming among weaker students and offering tailored recommendations is particularly relevant for educational institutions aiming to enhance learning outcomes.

Response 1: Thanks to the reviewer for the pleasant evaluation of the manuscript.

Comment 2: Weakness and Recommendations

(a) Weakness: Dataset Limitations: The study is limited to data from Computer Science and Engineering students from various universities in Bangladesh, which may not generalize to other contexts or educational settings.

Recommendation: Expand Dataset Scope: Future studies could explore the applicability of the proposed system across a broader range of disciplines and educational contexts to enhance the generalizability of the findings.

(b) Weakness: Complexity of Implementation: The manuscript does not fully address the practical challenges of implementing such an advanced system across different educational platforms or the training required for educators to effectively utilize this technology.

Recommendation: Implementation Guide: Providing a detailed guideline on implementing and integrating this EDM system into existing educational infrastructures could increase its practical utility.

(c) Weakness: Lack of Comparative Analysis: While the manuscript discusses the superiority of the proposed method over existing techniques, it lacks a direct comparison with other state-of-the-art EDM systems outside the scope of the presented literature.

Recommendation: Comparative Performance Study: Conducting comparative studies with other advanced EDM systems in real-world educational settings could further validate the effectiveness of the proposed approach.

Response 2: Thanks for pointing out the insightful weaknesses of our work and providing the corresponding recommendations to mitigate them. We have incorporated your recommendations as follows:

(a) As per the reviewer's suggestion, we have now updated the future work part of the revised manuscript (**Section 6, page#19-20**) to enhance the generalizability of the findings and to explore the applicability of the proposed system across a broader range of disciplines and educational contexts.

(b) Following the reviewer's recommendation, we have now added the new **Section 5.3 (page#19)** in the revised manuscript to include a detailed implementation guideline for the proposed system in educational settings.

(c) Thank you for your valuable feedback regarding the comparative analysis. We understand the importance of validating our proposed method against other EDM systems in real-world educational settings. Please notice that our EDM approach integrates XAI techniques with customized algorithmic enhancements specifically tailored for

programming skill assessment and enhancement contexts. Due to this unique integration, we did not find any directly comparable EDM systems in the current literature. However, in lieu of direct comparisons, we have compared our EDM system with the most relevant existing approaches in the literature, where the results of the proposed and existing EDM approaches are shown in **Table 1 (page#3)** of the manuscript. These include traditional machine learning models and other advanced techniques used in similar educational settings. We have benchmarked our results against widely accepted standards and performance metrics in the field of EDM to demonstrate the effectiveness and robustness of our approach. The results of these comparisons and benchmarks have been added to the manuscript, specifically in **Sections 4 and 5 and Tables 5 to 15**. We have also provided a detailed discussion of how our approach stands out in terms of interpretability, accuracy, and practical applicability. In addition, we have included a discussion on potential future work (**Section 6, page#19-20**), which could involve direct comparisons with more advanced EDM systems incorporating similar techniques as they become available.

To Reviewer 3

Comment 1: This paper introduces an advanced educational data mining (EDM) system for classification and improving programming skills of higher education students. This method emphasizes effective feature engineering, appropriate classification techniques, and the integration of explainable artificial intelligence (XAI) to elucidate model decisions. Through rigorous experiments, including ablation studies and evaluations of six machine learning algorithms, a novel ensemble method, Stacking SRDA, was introduced, which performed excellently in accuracy, precision, recall, F1 score, ROC curve, and McMahon test. The use of XAI tools provides insights into the interpretability of models. In addition, a system has been proposed to identify skill gaps in programming, providing customized skill enhancement suggestions for weaker students. The system seems very promising as it combines the latest technologies of Educational Data Mining (EDM) and Interpretable Artificial Intelligence (XAI), emphasizing effective feature engineering and appropriate classification techniques, which are necessary for establishing accurate predictive models. In addition, the system also utilizes XAI tools to provide interpretability of the model, thereby enhancing the understanding of model decisions. However, I believe that some parts of this paper still need to be revised, and I will provide my opinions from both the content and structure of the paper.

Response 1: Thanks to the reviewer for the valuable insights.

Comment 2: Dataset Description: The paper did not provide a detailed description of the dataset used, such as its source, size, characteristics, and preprocessing steps.

Response 2: Thanks to the reviewer for providing the feedback. We have now provided more descriptions of the dataset in **Section 3.2** of the revised manuscript. In addition, please refer to **Table 2**, which contains all features and their value levels in the dataset.

Comment 3: Reasonability of Algorithm Selection: Is the ML algorithm selected in the study the most suitable for solving the problem of predicting student programming performance? I need to know the answer to this question.

Response 3: From the literature review (**Section 2**), our findings demonstrate that existing EDM works commonly use the ML algorithms we employed in our experiment to predict student performance. Additionally, we proposed an ensemble learning algorithm, called Stacking-SRDA, to further enhance classification performance. However, we realized that our previous manuscript overlooked the inclusion of the Logistic Regression (LR) ML approach in the experimental analysis. Consequently, we have now incorporated LR, theoretically described in **Section 3.4**, and its experimental performance in **Section 4** of the revised manuscript.

Comment 4: Selection of evaluation indicators: I think that the evaluation indicators in the paper (such as accuracy, precision, recall, F1 score, etc.) are not sufficient to comprehensively evaluate the performance of the model. The author can try to find other evaluation indicators or measurement methods that are more suitable for this task.

Response 4: We appreciate the reviewer's thoughtful inspection. We have now incorporated two more evaluation indicators (RMSE and Cohen Kappa), theoretically described in **Section 3.5 (page#9-10)**. Please refer to the updated results presented in **Section 4** of the revised manuscript.

Comment 5: Consistency in interpretation of results: The authors need to provide an accurate explanation for the performance improvement of the model in the article. For example, why can the application of SMOTE and NearMss techniques improve the performance of the model?

Response 5: We sincerely appreciate the insightful feedback of the reviewer. In response to the reviewer's suggestions, we have now added the explanation for the performance improvement of the model in the last paragraph of **Section 4.3 (page#13-14)** of the revised manuscript.

Comment 6: Structure

(a) Title accuracy: The title can be modified to more clearly summarize the purpose and focus of the research.

(b) Experimental Design Description: The experimental design needs to clearly describe how to perform data preprocessing, model training, and performance evaluation, providing sufficient details for other researchers to replicate the experiment.

Response 6: (a) Thanks for the constructive suggestion. We have updated the title of the manuscript to "Enhancing Tertiary Students' Programming Skills with an Explainable Educational Data Mining Approach" following the reviewer's guidelines.

(b) We have double-checked the manuscript to ensure that **Sections 3 and 4** include in-detail descriptions of the dataset and clearly describe how to perform data preprocessing, model training, and performance evaluation to replicate the experiment.

To Reviewer 4

Comment 1: Your article could be really interesting, but in my opinion suffers from a substantial problem. You have chosen to compare many ML algorithms with a simple train-test split, so any numbers you provide may NOT be significant. The only sound method to compare ML algorithms is Cross Validation (and sometimes Repeated Cross Validation).

Response 1: We sincerely appreciate your thoughtful inspection. In response to the suggestion, we have now employed 10-fold cross-validation for all ML algorithms used in our study. Please refer to the newly added **Section 4.5 (page#15) and Table 13 (page#15)** to get this updated result analysis in the revised manuscript.

Comment 2: Every test that you made must be expressed in term of mean(metrics) +- std(metrics), otherwise your conclusion can be flawed by the random 80-20 (or whatever ratio) choice, that can be a REALLY influential choice (without your knowledge).You can see an example of the right approach in the python library PYCARET.

Response 2: We sincerely appreciate the constructive feedback. In response to the reviewer's suggestions, we have now updated all the experimental results to represent them with the mean value of the trial experiments, and we have also calculated the standard deviation for the five trials. Please refer to the experimental results presented in **Section 4** of the revised manuscript.

Comment 3: It is quite obvious that changing the train test ratio from 80-20 to 50-50 decreases performance, all else being equal: the algorithm has less data to train and generalizes worse. Thus, the point of conducting this test is not clear.

Response 3: Thanks to the reviewer for inquiring for more clarification on the retention of 50-50 training-testing ratio results in the manuscript. By altering the train-test ratio from the conventional 80-20 split to the 50-50 split, we sought to explore how the proportion of training data affects the proposed ensemble model's accuracy in comparison to the classical ML models. This investigation aims to shed light on the data requirements necessary for the ML models to maintain reasonable performance levels. Consequently, through this experimentation, we aim to provide insights that will help developers better understand the data dependencies of their models, thereby facilitating more effective and efficient model development strategies. We have now comprehensively incorporated the above discussion in **Section 4.2 (page#12)** of the revised manuscript.

Comment 4: It is also not clear why you choose LIME instead of SHAP for the local explainability.

Response 4: Thanks to the reviewer for the query. Both LIME and SHAP can be used for local explainability. Depending on our dataset and models with easily interpretable visualizations as well as to generate separate explanations for each class, we use LIME as it is useful and faster for simpler models and smaller datasets. In addition, given our system's design and the tools we were using, LIME's integration process was straightforward, ensuring a smooth implementation without significant modifications to our architecture. **Fig. 6 to Fig. 9** represent the local explainability using LIME, where we discussed the weight or importance of the features for individual classes. As such, this approach provides clear, simple, and easy-to-understand explanations, which is particularly

beneficial for educators and students involved in our presented EDM system who may not have a deep technical background. We have now comprehensively incorporated the above discussion in **Section 5.2 (page#17-18)** of the revised manuscript.
